# Differences in clinical knowledge levels between residents in two post‐graduate rotation programmes in Japan

**DOI:** 10.1186/s12909-021-02651-6

**Published:** 2021-04-21

**Authors:** Saki Muroya, Sachiko Ohde, Osamu Takahashi, Joshua Jacobs, Tsuguya Fukui

**Affiliations:** 1 Graduate School of Public Health, St. Luke’s International University, OMURA Susumu & Mieko Memorial, St. Luke’s Center for Clinical, Academia 5th Floor, 3-6 Tsukiji, Chuo-ku, 104-0045 Tokyo, Japan; 2 Sakakibara Heart Institute, 3-16-1 Asahi-cho, Fuchu-shi, 183-0003 Tokyo, Japan; 3 Department of General Internal Medicine, St. Luke’s International Hospital, 9-1 Akashi-cho, Chuo-ku, 104-8560 Tokyo, Japan; 4 Department of Medical Education and Clinical Sciences, Elson S. Floyd College of Medicine, Washington State University, 412 E. Spokane Falls Blvd, WA 99202-2131 Spokane, USA

**Keywords:** Japanese residency education, Post-graduate training, Clinical knowledge, Clinical competency, PLAB test

## Abstract

**Background:**

In Japan, between 2010 and 2020, there were two post-graduate training curricula for post-graduate medical education, as follows: comprehensive rotation programmes (CRPs), which require rotation in at least seven clinical departments; and limited rotation programmes (LRPs), which require rotation in fewer clinical departments. The curriculum that should be used for standardized Japanese post-graduate training has long been debated. Multiple studies show that post-graduate trainees who trained with CRPs were more satisfied and confident and gained more clinical experience than those who trained with LRPs. However, a comparison of objective measurements of the clinical knowledge of Japanese post-graduate trainees has not been reported. The aim of this study is to objectively measure and compare the clinical knowledge of trainees in CRPs and LRPs using a component of the Professional and Linguistic Assessment Board test (PLAB test).

**Methods:**

A nationwide cross-sectional study was conducted in February and March 2020. Post-graduate trainees who graduated from medical school were voluntarily recruited from general hospitals in Japan. To objectively measure the trainees’ basic clinical knowledge, the PLAB test was adapted from the UK. The cut-off point was set at 63%, as recommended by the UK General Medical Council. A statistical analysis was conducted to determine whether post-graduate programme differences contributed to the trainees’ test scores.

**Results:**

Twenty-two facilities volunteered to participate after recruitment, and 97 trainees from 19 facilities participated in the study. Thirty-one participants (32%) were in a CRP, and 66 participants (68%) were in an LRP. According to multiple logistic regression, the adjusted odds ratio of CRP trainees being in the high-scoring group was 5.16 (95% CI: 1.28-20.73, *p*<0.05). Mean differences in the scores in paediatrics, mental health and neurology were statistically higher among CRP trainees than LRP trainees.

**Conclusion:**

Post-graduate trainees who were in a CRP had better basic clinical competence knowledge (PLAB test) scores and performed better when tested in a wider range of subspecialties. Not only exam performance but also clinical performance and the longitudinal trend of trainees’ competency in post-graduate medical training should be evaluated in future studies.

## Background

In the late 19th century, Sir William Osler and William Stewart Halsted proposed the importance of hospital-based medical training for young medical students prior to their advanced training in a medical or surgical specialty [1]. Currently, clinical rotation training is implemented in many countries to allow student doctors or newly qualified doctors to gain additional practical experience in clinical skills through engagement with various clinical cases under the supervision of attending doctors. In the United States, student doctors at medical schools are most commonly educated in the specialties of internal medicine, surgery, paediatrics, psychiatry, obstetrics and gynaecology, family medicine, radiology, and neurology before graduation. Most accredited residency programmes require post-graduateyear-1 trainees to complete training in multiple specialties prior to completing the final examination of the United States Medical Licensing Examination (USMLE Step 3), which is required for licensure in the U.S. In the United Kingdom, post-graduate trainees are required to complete a foundation programme and have opportunities to experience an array of specialties and healthcare settings [2]. In Australia, similar to the U.S. and elsewhere, medical students are often considered a part of clinical teams and operate under the instruction of post-graduate trainees and attending physicians to develop their clinical skills to a level that is appropriate for initiating training as first-yearpost-graduate trainees. After graduation, medical students are required to undertake rotations in medicine, surgery, and emergency medicine [3]. As in other countries, early exposure to various medical specialties and clinical settings is common practice in the training of medical doctors.

In Japan, two-yearpost-graduate training after graduation from medical school is the only opportunity young doctors have to learn basic clinical skills in multiple specialties prior to progressing to advanced specialty training. This two-yearpost-graduate training is considered equivalent to the post-graduate year one (“internship”) of American medical graduates or the foundation programmes in the UK, where trainees rotate through numerous departments [4]. When the standardized post-graduate training programme was first introduced nationwide in 2004 in Japan to ensure the quality of licensed medical doctors, all post-graduate trainees were required to train in a comprehensive array of clinical specialties through comprehensive rotation programmes (CRPs) that included internal medicine, surgery, emergency, obstetrics-gynaecology, paediatrics, psychiatry, and rural medicine. However, after the first revision of the programme in 2010, the limited rotation programme (LRP), which requires rotation in fewer clinical departments, was approved. As of March 2020, these two types of programmes have existed in hospitals in Japan [5].

Discussions regarding whether the rotation requirements of the CRP are better than those of the LRP in training clinically competent doctors are ongoing. Nomura et al. [6] reported that post-graduate trainees at university hospitals, which mainly provide the LRP, were less satisfied with the training system and clinical skills training than those at non-university hospitals, which provide the CRP. Ohde et al. [7] found that post-graduate trainees in LRPs showed less confidence and less case experience than those in CRPs, especially with respect to paediatrics and obstetrics/gynaecology, which are often excluded from LRPs in many hospitals. Nevertheless, these studies analysed subjective self-evaluation reports by trainees rather than objective measures of knowledge or performance. Therefore, the purpose of this study is to objectively measure and compare the clinical knowledge of post-graduate trainees in CRPs and LRPs. There are no national standardized mandatory exams of clinical knowledge upon PGY2 for post-graduate trainees in Japan.

The Professional and Linguistic Assessment Board (PLAB) test is an objective examination of post-graduate clinical knowledge used in the UK. The PLAB test is run by the General Medical Council (GMC), and consists of a written exam and an objective structured clinical exam (OSCE). In the UK, passing the PLAB test is required for medical doctors who qualified abroad to help ensure that they have adequate knowledge and skills to practice medicine in the region. The PLAB test is designed for doctors from other countries to prove that their clinical competence level is equivalent to that of UK graduates who completed their first clinical training.

The purpose of this study is to investigate the difference in clinical knowledge between residents in CRP and LRP using the Professional and Linguistic Assessment Board (PLAB) test for Japanese residents who completed their first clinical training.

## Methods

### Study design and population

 A nationwide cross-sectional sample of post-graduate trainees who graduated from medical school within the last two years (Post-Graduate Year 1 (PGY1) – PGY2) was recruited to participate in the study in February and March 2020 in Japan. The PLAB test from the UK was used in this study to measure the post-graduate trainees’ basic clinical knowledge.

With permission from the UK GMC, only the written exam component, which was translated into Japanese, was used in our study. We first translated 200 questions provided by the GMC from English into Japanese, back-translated the questions from Japanese to English, and validated the questions with practicing physicians. During this process, 20 questions were excluded as the authors determined that they were not relevant to the Japanese healthcare environment. Then, three Japanese medical doctors, 2 internists and 1 emergency medicine doctor inspected the content of each question and adjusted the items to reflect the Japanese medical practice context; the adjustments included corrections to the units of blood exams (mmol/L to mg/dL), questions regarding unapproved drug usage (such as medical marijuana in the UK), and questions regarding access to medical services, such as general practitioners in the UK. Finally, the written exam included 180 questions from 23 field of specialties and was implemented for 120 min under the monitoring of proctors. The cut-off point for passing the test was set at 63 % of the total score, which is the same cut-off point used for the original written component of the PLAB test in the UK [8].

We classified the resident rotation programmes into two groups, namely, the CRP group and the LRP group, using the Residency Electronic Information System (REIS) website (https://www.iradis.mhlw.go.jp/reis/common/ad0.xhtml), where detailed information regarding the resident programmes is publicly available. We defined the programmes as comprehensive rotation programmes (CRPs) if the programmes included rotation in internal medicine, surgery, emergency, obstetrics-gynaecology, paediatrics, psychiatry, and rural medicine. To increase the generalizability of the study results, we attempted to recruit post-graduate trainees from as many hospitals as possible. University hospitals were excluded because they usually train numerous trainees and could occupy the entire LRP group. The authors asked for the cooperation of the Japan Organization of Advancing Medical Education Program (JAMEP) office to advertise and recruit post-graduate trainees for our study. The JAMEP conducts a voluntary examination called the General Medicine In-Training Examination (GM-ITE) for post-graduate trainees in Japan [9]. The JAMEP sent the invitation letter for the PLAB test to 211 institutions in Japan with GM-ITE information. Participation in the PLAB test was also voluntary, and all participants provided written informed consent prior to starting the exam. The study participants completed the exam in a proctored environment at their training site. The participants were compensated with a JPY 3,000 (approximately USD$30) gift card. After the data collection, the participants were anonymized for the subsequent analyses.

 The study protocol was developed according to the ethical guidelines of St. Luke’s International Hospital. Ethical approval to conduct the study was obtained from the Research Ethics Committee of St. Luke’s International Hospital, Tokyo, Japan (approval code: 18-R186).

### Statistical analysis

The test scores were calculated as 1 point per question and were converted to a 100-point scale. The participants’ scores were divided into the following two groups: a high-score group with scores ≥ 63 % and a low-score group with scores < 63 %. The significance level was set at less than 0.05, and all statistical tests were two-tailed. A multivariate logistic regression analysis was used to compare the test results between the CRP and LRP groups. In the secondary analysis, a comparison of the scores on each question was performed to determine whether there were any differences between the CRP and LRP groups by the type of question and subspecialty using a multiple linear regression. The structure of the PLAB test is shown in Table 1. The data were analysed using STATA statistics software version 13.0 (StataCorp LLC, College Station, TX, USA).

**Table 1:** Question categories in the PLAB test

Structure of the PLAB test	N (%)
Type of question	
Applying knowledge and experience to clinical practice	23 (12.8)
Good clinical care: assessment	108 (60.0)
Good clinical care: management	49 (27.2)
Total	180
Subspecialty areas	
Infectious disease	17 (9.4)
Cardiology	15 (8.3)
Mental health	15 (8.3)
Urology	13 (7.2)
Gastroenterology	12 (6.7)
Endocrinology	12 (6.7)
Reproductive health	10 (5.6)
Critical care	10 (5.6)
Orthopaedics	8 (4.4)
Neurology	8 (4.4)
Respiratory	8 (4.4)
Genitourinary	7 (3.9)
Dermatology	7 (3.9)
Ophthalmology	6 (3.3)
Breast surgery	5 (2.8)
Paediatrics	5 (2.8)
Otorhinolaryngology	5 (2.8)
Nephrology	5 (2.8)
Haematology	4 (2.2)
Pharmacology	4 (2.2)
Geriatrics	2 (1.1)
Medical ethics	1 (0.6)
Physiology	1 (0.6)
Total	180

## Results

 A final sample of 97 subjects from 19 facilities participated in the study. Three facilities withdrew participation prior to the study due to the COVID-19 pandemic.

Table 2 shows the demographic data of all study participants and each programme (LRP and CRP). The mean age was 26.9 (SD, 1.7) years; 74 participants (76.3 %) were male. Thirty-one participants (32.0 %) were in a CRP, and 66 participants (68.0 %) were in an LRP. Of the participants in an LRP, 28 participants from 8 facilities were required to receive training only in internal medicine, emergency medicine, and rural medicine, and 38 participants from 3 facilities were required to receive training only in internal medicine, surgery, emergency medicine and rural medicine. The mean test score of all participants was 66.5 % (SD, 6.6). The mean test score of the participants in a CRP was 68.4 % (SD, 5.7), and the mean test score of the participants in an LRP was 65.6 % (SD, 6.9). The mean difference between the participants in a CRP and those in an LRP was not statistically significant (*p* = 0.057). Table 3 shows the comparison of the high-score group and low-score group. The number of participants in a CRP in the high-score group was 28 (39.4 %), and the number of participants in the low-score group was 3 (11.5 %); this difference between the two programmes was statistically significant (*p* < 0.01).

**Table 2:** Demographic data of all study participants and each programme (LRP and CRP)

	Total (*N* = 97)	LRP* (*N* = 66)	CRP* (*N* = 31)	*p*-value
Age, mean (SD)	26.9 (1.7)	26.8 (1.7)	26.9 (1.8)	0.885
Sex (male), N (%)	74 (76.3)	49 (74.2)	25 (80.7)	0.478
Number of hospital beds (< 500)	76 (78.4)	53 (80.3)	23 (74.2)	0.496
(500≤)	21 (21.7)	13 (19.7)	8 (25.8)	
Total score (%), mean (SD)	66.5 (6.6)	65.6 (6.9)	68.4 (5.7)	0.057
High score group, N (%)	71 (73.2)	43 (65.2)	28 (90.3)	*p* < 0.01

Significance level: *p* < 0.05

*LRP limited rotation programme, *CRP* comprehensive rotation programme

**Table 3:** Comparison of the high-score group and low-score group

	High-score group (*N* = 71)	Low-score group (*N* = 26)	*p*-value
Age, mean (SD)	26.9 (1.8)	26.8 (1.4)	0.946
Sex (male), N (%)	56 (78.9)	18 (69.2)	0.323
Programme (CRP*), N (%)	28 (39.4)	3 (11.5)	*p* < 0.01
Number of hospital beds, mean (SD)	502.0 (173.4)	554.3 (144.0)	0.173
Total score (%), mean (SD)	69.7 (3.9)	57.9 (4.7)	*p* < 0.001

Significance level: *p* < 0.05

*CRP comprehensive rotation programme

Table 4 shows the results of the multiple logistic regression conducted to determine whether the post-graduate training programme is strongly associated with a PLAB test high-score result. After adjusting for age, sex, and number of hospital beds, the residents in a CRP were 5.26 times more likely to be in the high-score group than those in an LRP (*p* < 0.05, 95 % CI: 1.33–20.74). No other variables were statistically significant.

**Table 4:** Multiple logistic regression model of the high-score group

Variables	Adjusted OR	95 % CI	*p*-value
Age	0.99	0.75–1.33	0.993
Sex (male)	1.64	0.57–4.74	0.363
Number of hospital beds	1.00	0.99–1.00	0.154
Programme (CRP*)	5.26	1.33–20.74	*p* < 0.05

Significance level: *p* < 0.05

*CRP comprehensive rotation programme

Based on the secondary analysis, Table 5 shows the results of the multiple linear regression conducted to determine whether there were programme differences by type of question and subspecialty areas. The CRP post-graduate trainees showed significantly better knowledge of paediatrics (*p* < 0.01), mental health (*p* < 0.05), and neurology (*p* < 0.05) than the LRP trainees after adjusting for age, sex and number of hospital beds

**Table 5:** Multivariate linear regression analysis of the type of question and subspecialty areas

Question categories	β*	95 % CI	*p*-value
Type of question			
Applying knowledge and experience to clinical practice	2.55	-2.31–7.41	0.300
Good clinical care: assessment	1.25	-2.56–5.06	0.517
Good clinical care: management	1.79	-1.45–5.03	0.275
Subspecialty area			
Infectious disease	0.94	-3.76–5.64	0.692
Cardiology	3.38	-2.17–8.92	0.230
Mental health	6.48	1.34–11.61	*p* < 0.05
Urology	0.55	-4.61–5.70	0.834
Gastroenterology	1.63	-3.88–7.15	0.558
Endocrinology	0.89	-4.77–6.56	0.755
Reproductive health	0.17	-6.73–7.07	0.961
Critical care	-0.30	-7.27–6.68	0.933
Orthopaedics	3.46	-2.78–9.71	0.274
Neurology	8.56	0.77–16.35	*p* < 0.05
Respiratory	-2.94	-9.82–3.93	0.397
Genitourinary	5.17	-2.50–12.84	0.184
Dermatology	-1.23	-9.16–6.71	0.759
Ophthalmology	0.21	-7.59–8.01	0.957
Breast surgery	1.28	-7.56–10.12	0.774
Paediatrics	10.07	3.05–17.10	*p* < 0.01
Otorhinolaryngology	5.97	-0.89–12.82	0.087
Nephrology	5.75	-4.18–15.67	0.253
Haematology	6.54	-1.57–14.65	0.113
Pharmacology	2.59	-4.91–10.09	0.494
Geriatrics	5.52	-6.04–17.07	0.345
Medical ethics	-8.74	-25.38–7.90	0.300
Physiology	1.30	-10.73–13.34	0.830

Significance level: *p* < 0.05

*Outcome: continuous value of scores

**β is the adjusted mean score among CRP trainees

*β is the adjusted means score among CRP trainees, adjusted for age, sex and number of hospital beds

## Discussion

This study shows that the post-graduate trainees who participated in comprehensive rotation programmes (CRPs) had higher scores on an objective clinical knowledge test than the trainees who participated in limited rotation programmes (LRPs). The multiple logistic regression models also showed a significant difference between the training programmes after adjusting for age, sex, and number of hospital beds. In particular, the CRP trainees were more likely to exhibit a better understanding of paediatrics, mental health, and neurology. In a previous study, we reported that trainees in LRPs showed lower confidence and less case experience than those in CRPs [7]. A limitation of our previous study was that we relied on self-reported measures to investigate clinical knowledge. In the present study, this issue was addressed by administering a written test with validated items (a component of the PLAB test), and the trainees in a CRP still showed higher clinical knowledge scores than those in an LRP. Moreover, the trainees in a CRP showed higher scores in paediatrics and mental health, and LRPs often exclude these rotations.

In a rapidly aging society such as Japan, the disease burden experienced by patients is complicated by multiple chronic diseases, and clinicians are required to be able to provide holistic medical care to patients that includes areas outside of their specific subspecialty. The Ministry of Health, Labour and Welfare has been promoting the development of a community-based comprehensive care system in each community [10]. In this system, the integration of medical care, long-term care, public health, and welfare are necessary. Hence, doctors whose general specialty is internal medicine, surgery, etc. are expected to play a key role in developing a community that can serve the entire population from children to the elderly and from healthy residents to residents with illness or disability [11]. Primary care physicians are expected to respond to any acute/chronic health problem regardless of age, sex or disease/illness and be able to consider their patients’ physical, psychological, social, and cultural needs [12]. The development of medical education to produce medical doctors that can meet these patients’ individual and social needs is essential in Japanese society. The possibility of contributing to meeting these needs by providing a comprehensive array of clinical training is significant.

Globally, post-graduate medical education programmes tend to integrate multidisciplinary training, and multiple reports investigating whether early practical training in rotation programmes for medical students or post-graduate trainees may be associated with knowledge, attitude towards patients, confidence in delivering medical care, and clinical performance have been published [13–18]. Post-graduate training in Japan has undergone a drastic change since April 1, 2020 as LRPs have been entirely discarded, and all current programmes provide a comprehensive array of clinical training to post-graduate trainees before they begin their advanced clinical training (PGY3 and beyond) [19, 20]. The findings of the current study provide further evidence supporting this decision.

### Limitations of this study

Although the subjects were recruited in a nationwide effort, the number of facilities and participants was limited. One substantial burden during this study involved the competing priorities caused by the COVID-19 outbreak while recruiting the participants and administering the exams. While approximately 9,000 new graduate doctors participate in the post-graduate training system each year in Japan, we could only recruit 97 residents. Thus, the study results may be limited, and full representativeness cannot be guaranteed. Additionally, the small sample size contributed to the wide range of the 95 % confidence interval in the analysis.

Another limitation was that this study examined only the adequacy of the participants’ content knowledge. The original PLAB test consists of a written exam and an objective structured clinical exam (OSCE) to evaluate the clinical competency of foreign licensed doctors in the UK. The current study did not examine overall clinical competency or clinical skills. Furthermore, since medical education curricula are usually designed in relation to country-specific clinical exams, the test scores of Japanese post-graduate trainees may not be comparable to the test scores in the UK. Medical knowledge is not the only desirable competency for medical students and medical doctors. For example, the Accreditation Council for Graduate Medical Education specifies the following six competencies: patient care, medical knowledge, practice-based learning and improvement, professionalism, interpersonal skills and communication, and systems-based practice [21]. Our purpose of objectively comparing CRP and LRP trainees’ clinical knowledge was achieved in this study; however, to capture the clinical performance of post-graduate trainees, a clinical evaluation that measures additional clinical competencies could provide useful information to help inform policy decisions regarding post-graduate medical education.

## Conclusions

Residents who were in a CRP had better scores on the objective basic clinical knowledge test (a component of the PLAB test) and performed better when tested in a wider range of subspecialties than those in an LRP. In April 2020, post-graduate medical training was adjusted to include only the CRP curriculum. Therefore, both exam performance and clinical performance based on evaluations by clinical supervisors and patient outcomes should be examined, and longitudinal evaluations of all competency domains should be performed in the future to help inform the continued adjustment and refinement of post-graduate medical education in Japan.

## Data Availability

The datasets generated and/or analysed during the current study are not publicly available to protect the originality of our work such that we can continue evaluating future examinees but are available from the corresponding author upon reasonable request.

## Abbreviations

CRP/CRPs: Comprehensive rotation programme(s)
LRP/LRPs: limited rotation programme(s)
PLAB: Professional and Linguistic Assessment Board
